# Black Perinatal Mental Health: Prioritizing Maternal Mental Health to Optimize Infant Health and Wellness

**DOI:** 10.3389/fpsyt.2022.807235

**Published:** 2022-04-29

**Authors:** Tracey Estriplet, Isabel Morgan, Kelly Davis, Joia Crear Perry, Kay Matthews

**Affiliations:** ^1^National Birth Equity Collaborative, New Orleans, LA, United States; ^2^Shades of Blue Project, Houston, TX, United States

**Keywords:** perinatal mental health, inequaities, Reproductive Justice (RJ), birth equity, infant mental health, inequities in health care

## Abstract

Infant mental health is interconnected with and affected by maternal mental health. A mother or birthing person's mental health before and during pregnancy and the postpartum period is essential for a child's development. During the first year of life, infants require emotional attachment and bonding to strive. Perinatal mood disorders are likely to hinder attachment and are associated with an increased risk of adverse mental health effects for children later in life. The Black community is faced with a crisis as Black mothers experience a higher prevalence of perinatal mood disorders, including postpartum depression and anxiety, compared to the United States national estimates. The aim of the research is to identify social, structural, and economic disparities of Black perinatal women and birthing people's experience to understand the impact of perinatal mental health on infants' mental health. Black mothers and birthing people may often face social and structural barriers that limit their opportunity to seek and engage with interventions and treatment that address the root causes of their perinatal mood disorder. To enhance understanding of racial disparities caused by social and structural determinants of health on Black mothers and birthing people's mental health and health care experiences that influence infant mental health, the study team conducted semi-structured interviews among self-identified cisgender Black women health professionals nationwide, who provide care to pregnant or postpartum Black women and birthing people. Our study attempted to identify themes, pathways, interventions, and strategies to promote equitable and anti-racist maternal and infant mental health care. Using a Rigorous and Accelerated Data Reduction (Radar) technique and a deductive qualitative analytic approach it was found that limited access to resources, lack of universal screening and mental health education, and the disjointed healthcare system serves as barriers, contribute to mental health issues, and put Black mothers and birthing people at a disadvantage in autonomous decision making. Our study concluded that instituting education on healthy and culturally appropriate ways to support infant development in parent education programs may support Black parents in establishing healthy attachment and bonds. Prioritizing strategies to improve maternal mental health and centering Black parents in developing these educational parenting programs may optimize parenting experiences.

## Introduction

An estimated 10–20% of perinatal depressive episodes meet the full criteria for a major depressive episode (1). The prevalence of perinatal depression increases to 25–50% when minor bouts of depression or anxiety are included (2). Untreated perinatal depression and anxiety are associated with pregnancy complications and adverse outcomes during the perinatal period, including impaired lactation, suicide, and infanticide (3). Black mothers experience a higher prevalence of maternal mental health issues, including postpartum depression and anxiety, in comparison to the United States national estimates (4).

There is an interconnectedness between the mental and emotional health of the person giving birth and the developing fetus. A mother or birthing person's mental health before and during pregnancy and the postpartum period is essential for the development of a child (5). During the first year of life, infants require emotional attachment and bonding to thrive. The bond that is formed during this stage is the foundation on which young children develop self-awareness relative to others (6). Mothers who experience depression or anxiety and lack of social support may find it challenging to build a secure attachment, leading to an insecure attachment (3) that can impact an infant's capacity to regulate their emotions and behaviors, learning, and assessment of social cues (7). Maternal mental health disorders such as depression, anxiety and psychosis are also associated with an increased risk of adverse mental health effects for children later in life (8).

To understand the root causes of maternal and infant mental and emotional health, it is imperative to use the frameworks of social justice movements, like Reproductive Justice and birth equity, that use power analyses to contextualize the social and structural determinants of maternal, infant, and reproductive health. Loretta Ross, co-creator of the reproductive justice movement, defines reproductive justice as, “the human right to maintain personal bodily autonomy, have children, not have children, and parent the children we have in safe and sustainable communities” (9). The birth equity framework focuses on rectifying structural racism and social determinants through system-level change and initiatives to improve maternal and infant health (10).

Applying these frameworks helps address intersecting oppressions, and center marginalized populations in developing pathways to improve maternal mental health and its barriers to care and health equity. Specifically, intersecting social constructions such as gender and race and accompanying systems of oppression such as racism, misogyny and transphobia create barriers to pregnant and parenting families having what they need to thrive. To overcome the persistent racial inequities in perinatal mental health for better infant mental health outcomes, we must consider, in finding solutions, those who bear the greatest burden of structural racism and gender oppression – Black mothers and birthing people.

The experiences of Black mothers and birthing people is key to identifying viable solutions to improving maternal and infant mental health inequities. Limited access to resources, lack of universal screening and mental health education, lack of culturally congruent providers and fractured healthcare systems limit the ways Black mothers and birthing people often seek and receive health services (11). These social and structural barriers contribute to mental health issues and put Black mothers and birthing people at a disadvantage in autonomous decision making (11). To improve the awareness and understanding of the impacts of social and structural determinants of mental health for Black mothers, birthing people and their infants, we conducted semi-structured interviews with Black maternal and infant mental health stakeholders and experts.

This article reports pathways to address social and structural inequities that Black women and birthing people experience during the perinatal period, to identify pathways to equitable maternal mental healthcare to optimize infant health and cognitive development.

## Materials and Methods

### Study Team

The study team comprised of three cisgender, Black women, public health professionals. Two of the team members hold masters-level training in public health and had experience conducting qualitative research and analysis using analytic software. One member of the team was a trained licensed clinical health worker, with a specialization in providing mental health counseling and care to Black women.

### Study Setting

We conducted semi-structured interviews from February 16th, 2021, to March 10th, 2021, among self-identified cisgender Black women health professionals nationwide, who provide care to pregnant or postpartum Black women and birthing people.

### Grounding Frameworks

In developing the interview guides, principles of reproductive justice and birth equity informed the questions, data collection and analysis, and interpretation. The interview guides included specific questions regarding the impact of the mother's (or birthing person's) health on their infant's health and mental well-being. The guide also contained questions regarding the effects of systems of oppression (e.g., racism, gender oppression) and social and structural determinants of health on Black mother's access to mental health services, treatment, and their overall mental health status.

The Reproductive Justice (RJ) movement began in 1994 by Black feminist advocates. The RJ movement declares an individual's rights to bodily autonomy not to have children, the right to have children, including all birthing options, and to parent their children in safe and supportive communities (9). The RJ movement proposes a theoretical framework that explains how intersecting systems of oppression create the conditions that most often impact Black birthing people and their experience with the mental health care system, including maternal mental health providers and practitioners. Inequities in mental health service utilization and provision stem from the lack of incorporation of the Reproductive Justice (RJ) Framework into clinical practice. The RJ framework is a critical concept of reproductive autonomy that acknowledges how reproductive rights are adversely impacted by racism, paternalism, economic injustices, and other forms of oppression. At the intersection of mental health, education and autonomous rights, the availability for culturally congruent, unbiased, decolonized (10), trusted mental health providers is a useful quality indicator for pregnant and postpartum Black birthing people's use of mental health services. Further, RJ asserts that children deserve to be raised in safe and supportive communities, and that begins in the home and within the family unit.

Birth Equity is defined as “the assurance of the conditions of optimal births for all people with a willingness to address racial and social inequities in a sustained effort” (11). This framework explains how structural and social determinants impact perinatal health outcomes and the wellbeing of pregnant and postpartum populations.

The RJ and Birth Equity frameworks provide a lens of centering and valuing the autonomy and self-knowledge of people in marginalized communities within systems. These theoretical frameworks provide a basis for understanding not only how systems and structures impact perinatal mental health, but also the relationship between maternal mental health and infant mental health and wellbeing.

### Participants and Recruitment

This study was approved by the Institutional Review Board of the Institute for Women and Ethnic Studies on December 18th, 2020. The interview guide comprised 21 questions, organized in six major sections: Framing, Barriers, Racism and Gender Oppression, Social Determinants of Health, Solutions and Strategies, and Infant Mental Health (Exhibit Appendix 1). The interview guide has been published previously (12). Of the six sections, five focused on maternal mental health, while one section focused exclusively on infant mental health. Results from the analysis of data on maternal mental health have been published previously (12). This article presents an analysis of the data specific to infant mental health and wellbeing.

The study team developed a preliminary list of 40 stakeholders with expertise in maternal or infant mental health. The final stakeholders prioritized Black women who had expertise in reproductive justice or birth equity and infant or maternal mental health. The stakeholders who provided counseling services had to serve predominantly Black women and birthing people, to be invited to the study. Stakeholders were invited via email to participate in one-on-one interviews with members of the study team. Of the 40 identified stake holders, ten stakeholders were invited and accepted the invitation to interview. The stakeholders were self-identified cis-gendered Black women, ranging reproductive age.

### Stakeholder Interviews

We interviewed 10 perinatal mental health practitioners, researchers, and activists during February and March 2021. Qualitative interviews were conducted one-on-one with stakeholders to understand the impacts of structural and social determinants of health on Black birthing people's mental health and mental health care experiences to identify solutions that could advance maternal mental health care and infant mental health. The stakeholders interviewed had a wide range of training expertise that included perinatal social work, clinical psychology, social work, birth work, lactation support, marriage and family therapy, and health disparities research linking the mental health and wellbeing of infants. Several stakeholders provided services to pregnant and postpartum Black women and birthing people in hospital and outpatient settings. Stakeholders were asked a series of open-ended questions to determine the perceived barriers Black women and birthing people experience in maternal mental health systems of care.

All interviews were conducted virtually via Zoom Communications Technologies software. Interviews were video and audio recorded with the participant's consent. A second member of the study team participated in interviews as an observer to provide technical support, obtain verbal informed consent and take notes. Audio recordings of each interview were submitted to REV-a professional audio transcription service that uses humans to transcribe audio to limit inaccuracies of the interviews.

### Qualitative Analysis

The study team conducted a rapid and in-depth analysis to identify significant themes from the stakeholder interviews. The team utilized an iterative process for the rapid analysis to arrive at final themes from February to March 2021. The rapid analysis was guided by the Rigorous and Accelerated Data Reduction (RADaR) technique characterized by using tables and spreadsheets in Microsoft Excel to develop an all-inclusive data set that underwent multiple revisions to synthesize common themes among the interviews (13). Two team members created an excel spreadsheet with the facilitator's questions in rows and responses from each stakeholder in the columns. One team member created a document with pseudonyms and scopes of practice for each participant. Preliminary themes were presented to 13 perinatal mental health stakeholders during two virtual webinars in March 2021 to obtain feedback on the identified themes from the rapid analysis and inform the development of equitable and antiracist maternal and infant mental healthcare pathways. A flyer for the virtual webinar sessions was shared via social media channels of the author's affiliated organizations and disseminated by email through an organizational newsletter of one author's community-based maternal mental health organization. We captured insights from webinar participants verbally and through Jamboard to determine if the preliminary results from the rapid analysis of the stakeholder interviews resonated with them based on their experience and expertise. The webinars were co-facilitated by the study team and two Black maternal mental health professionals who participated in the interview series.

An in-depth analysis was then conducted using a deductive qualitative analytic approach. The preliminary codebook (Exhibit Appendix 2) was curated based on the initial grounding literature review, the guiding frameworks (Reproductive Justice and Birth Equity), and team members with maternal and infant mental health expertise to construct a foundation for analysis. Two team members independently coded two transcripts using the preliminary codebook and reviewed the coded themes for definition accuracy. The codebook was then revised to redefine and expand codes. The remainder of the transcripts were coded independently by two team members, then reviewed for accuracy and identification of themes. The themes were then discussed with the third team member who facilitated the stakeholder interviews to ensure that significant findings were consistent with interpretations of the webinar summaries and discussions.

## Results

Three themes specific to infant mental health were identified from interviews with the perinatal mental health stakeholders, including challenges conceptualizing and defining, relationship between maternal health and infant health and wellness, and strategies for nurturing infant health and wellness.

### Challenges of Conceptualizing and Defining Infant Mental Health

Participants emphasized that infant mental health is challenging to conceptualize because it lacks an operational and cohesive definition. In describing how infant mental health is defined, one practitioner shared:

“I got asked to be a speaker for an infant mental health organization, and I was like, can you describe to me what that means to you? I don't know exactly what that means… It's literally like a term that doesn't really have a full operational definition. I feel like, in the field, none of us are learning infant mental health, really. We might learn developmental health and then sort of the course of what infants might be doing in different stages, but not that there's a whole provider geared to work with zero to three in that way.” - Felicia, Licensed Clinical Psychologist and Birth Doula.

This participant acknowledges that there are providers whose scope of practice is dedicated to infants from newborns to 3 years old. While their clinical training included content focused on infant developmental health, the specific terminology and focus on infant mental health was not addressed. Another participant also expressed difficulty in specifying what infant mental health encompasses:

“[Infant mental health is] hard to capture, related to the environmental structure, but something that we can work on.” - Alesia, Health Disparities Researcher.

While the operational and cohesive definitions of infant mental health were unclear, practitioners noted that the scope of infant mental health encompasses an infant's cognitive and emotional development. Mother-infant bonding and attachment were often referred to as indicators of an infant's mental health status:

“Infant mental health to me means a provider that is aware of what the stages of the development should be, particularly in this early infant side, which is a lot about secure attachment.” - Felicia, Licensed Clinical Psychologist and Birth Doula.

“The first thing that comes to my mind is secure attachment, like are you doing what you need to do to provide a secure attachment with the parents and the child? Or even understanding the journey of your baby. Because people are like, ‘Oh, that baby's this and that.' Just understanding what babies are going through or young toddlers are going through to really help with that bond between parent and child.” Morgan, Licensed Mental Health Therapist.

One participant described the importance of acknowledging an infant as a separate being with their own needs and journeys. Related to understanding the spiritual journey of infants, one practitioner noted how our conceptualization of infant mental health has been medicalized:

“When we think about infant mental health, we think about it from a clinical standpoint or a biological, neurological standpoint. But then there is a spiritual piece to this, where there are traditions that recognize that just because someone is a baby doesn't mean that they don't have wisdom or that they're not an old soul, or that they're not an ancestor returned.” - Cassandra, Licensed Marriage and Family Therapist.

### Relationship Between Maternal Health and Infant Health and Wellness

While several participants expressed a lack of clarity regarding how to define infant mental health, all acknowledged that the environment impacts infant health and wellness, including the effects of health of the pregnant or postpartum person-including mental health-on the attachment, bonding, development, and mental health of an infant, as stated:

“What comes to mind, to me right away, is: What was going on while they were in the womb? And what was happening to their mom's body before she even conceived? What was being held up in her body, that is just going to pass right on to her little baby? So that's where infant mental health starts, not when [the infant is] born.” - Yvette, Birth and Postpartum Doula.

“Infant mental health is a conversation I feel like that should couple maternal health as well, because mothers need to know how their mental health stressors and the stressors of the environment may play a role in how that child develops in many different ways.” - Monica, Perinatal Social Worker.

One practitioner emphasized how the mental health of a pregnant or postpartum person extends beyond the home to impact the community.

“That whole thing is cyclical. If mama, parent's mental health isn't intact, that's going to have a manifestation into the children. That's going to have a manifestation into the community, that's full circle.” – Tayler, Certified Breastfeeding Specialist and Parenting Support Coach.

However, several participants expressed concerns about the blame and shame that Black mothers and birthing people experience related to perceptions about their parenting and the impact it has on the family dynamic, as stated by a licensed marriage and family therapist:

“If that mother doesn't recognize early enough, well, one, if she's not valued. If she's not valued, if she's not centered, or if her needs aren't met, they may try to meet the needs of this child, and they may find it difficult to do so, and that can lead to worth feelings and feelings of hopelessness. But more than that, if they don't see this as a co-created relationship where I can impact this child, I have an impact. See, this is the thing about power, if I'm disempowering this mother in the doctor's office, I'm disempowering this birthing person, then when they go home, how are they supposed to feel empowered about the relationship they're establishing with this child? How isolating can that be for both?” Cassandra, Licensed Marriage, and Family Therapist.

“So, at the same time with this research, a lot of it makes me sick to my stomach when reading the literature out there on infant mental health. A lot of it is mom shaming, mom blaming, fetal programming, the infant experience is destiny. You'll read articles on maternal depression and obesity, and maternal depression and offspring suicide. Like, ‘Well, it was all her fault.' So, there's a lot of work to be done there.” - Alesia, Health Disparities Researcher.

Similarly, one practitioner emphasized how Black mothers are central to Black family structures and described the added stressors that Black women experience in parenting with a mental health condition:

“The Black mom is the one that's making sure everything is going and happening and moving and shaking. And pretty much the Black mom could be dripping blood and people are still like, are we going to eat? Can I get my medicine? I want to talk to you about my bad day at work. And I also want you to still be emotionally available for the meddling mother-in-law and all that stuff. Not getting treatment for maternal mental health, it can really break down the Black family, because of Black family is really relying on that Black mom to advance the family and keep a lot of things going. Whereas, I see with my white moms, they're just ‘I'm crying',

‘I'm sad' and people need to get it together.” - Annette, Licensed Clinical Professional Counselor.

“So, one of my favorite questions that I ask all the parents that I work with is like, ‘How are you feeling about parenthood these days?' And that's just the opening question. It allows them the space to say like, ‘I don't know what the heck I'm doing. This baby crys all the time. I don't know the expectations that this kid have on me.' So, I allow them to vent, and then I say, ‘Okay, what are you able to do right now? Are you able to just play with that kid when they doing tummy time? Do you sing? Do you like to sing?' And I had one mom, she loved to sing, and she thought she couldn't sing to the child. I was like, Sing your gospel songs. Sing to the child. That's a connection, because the baby'll probably start singing back with you. And just helping parents use their strengths to connect with the child. You don't have to be this Instagram, Pinterest-worthy whatever. Use your strength. If you like to build, get some LEGOs for the kid, even if it's a baby. Just like, ‘Hey, look at this. It's yellow. It's red.' Use your strengths to connect with the child so it feels accessible. They don't have to do anything extra. They're using what they already have. And so I turn it to that way. ‘What do you already have in your house? I'm not saying you got to build something or spend money. Let's use what you already have to connect with the baby. The baby don't care. The baby just wants to spend time with you. The baby just wants to learn from you.' And just breaking it down that way so they feel like they do have the skills that they need to connect with their child.” -Morgan, Licensed Mental Health Therapist.

### Suggested Practical Interventions for Health Services and Healthcare Providers

Participants emphasized that Black mothers and birthing people should be prioritized, centered, and valued in the hospital setting and at home to provide a nurturing environment for their children.

“So, I mean, it just shows you that all of this is happening in the womb while they're *in utero*. And so, that's why you want so much for a mom to be mentally well even in those preconception years so that, baby can be well. - Yvette, Birth and Postpartum Doula.

Several participants described the importance of examining root causes, and identifying social and structural factors that impact the experiences and care of Black pregnant and postpartum people and their families.

“It's how we automatically believe folks are inadequate and disposable. It's so deeply rooted in our society.”

- Tayler, Certified Breastfeeding Specialist and Parenting Support Coach.

“Diversifying [the workforce] is one thing, but the reality is, we've all been socialized to uphold harm and oppression. That cultivation and socialization, even a lot of birthworkers who got training from white-led organizations, there's a lot of work that has to be done to decolonize and deconstruct how they have been taught. Same thing within the social sector within mental health and therapist, where literally your licensing is depending on you being harmful, toxic and oppressive and not knowing because you're so deeply rooted to that…Dr. Dorothy Roberts does a phenomenal job of looking at the connection to Child Protective Services (CPS) and it's connection to taking away families. How do you defund the systems that are connected to incarceration, that are connected to hyper-policing? It's not just the diversifying piece, we also have to have folks who are accountable and who have gone through the training and understanding.” - Tayler, Certified Breastfeeding Specialist and Parenting Support Coach.

“There's this overuse of being trauma-informed. How are you trauma-informed and healed but just knowing that people have trauma, but not acknowledging where that trauma has come from and the root causes of that trauma? How can you still cast blame on individuals and not systems?” - Tayler, Certified Breastfeeding Specialist and Parenting Support Coach.

It was theorized by one practitioner that teaching all parents the signs and symptoms of mental distress and lack of maternal-infant bonding, and also equipping them with resources would help mitigate the lack of attachment and bonding for parents who are faced with maternal or perinatal mental health issues:

“I think if you are working with mothers and birthing people and helping them understand how to self-regulate their own emotions, they will be more capable and more responsive to respond to this infant's needs, because if you're working on your anxiety and you notice, okay, well, this is what I do when I get anxious so let me take some deep breaths, then when the baby comes and there's a whole bunch of stuff happening, and you feel anxious, you respond to yourself in a healthy way, and you take a deep breath, and the children learn that the infants see that. You're not going to be perfect, but you're going to teach resilience to an infant.” - Yolanda, Licensed Clinical Psychologist.

Additionally, postpartum doulas and traditional healing practices were presented as potential solutions to support parents nurture healthy infant mental health by centering birthing people's healing postpartum:

“I think another resource that will be great, and then this goes back to the healthcare and health systems is having… If someone can't afford to have a postpartum doula come in and helping if you don't have family around. There are some insurances that will pay for a visiting nurse. But you usually get like one visit, but what if that was part of health care that you were provided with like that after care, that you had someone that was able to come in and again, do all the things so that you could really just be in that restful period, we talk about the 40 days. And those African traditions of mom is literally just in her bed, and everyone is bringing food to her, warming foods to her so that her body can heal, so that she can feed her baby and be able to love on her baby, without the mundane tasks of life.” - Yvette, Birth and Postpartum Doula.

Above all, all practitioners and participants identified addressing maternal mental health care as the number one solution to mitigate infant mental health distress and disorders. “In line with a focus on centering Black mothers and birthing people, practitioners identified five pathways to improve and access to equitable and antiracist mental health care”.

### Pathways Toward Equitable and Antiracist Maternal and Infant Mental Health Care

The five key pathways identified are (a) educating and training practitioners, (b) investing in Black women mental health workforce, (c) investing in Black women-led community-based organizations, (d) valuing, honoring, and investing in the community and, (e) promoting integrated care and shared decision making.

Practitioners emphasized these five pathways would equip both practitioners and Black mothers and birthing people with skills to adequately address social and structural challenges faced by the birthing woman or person, such as racism, medical mistrust, and race-based treatment plans. The impact of racism on the mental health workforce and access to pathways to mental health careers was an identified barrier. Racism was identified as the underlying cause of Black maternal health inequities, as Black women are underrepresented in the perinatal mental health workforce and among clients receiving mental health treatment. Additionally, to identify structural and social determinants of health areas of education and training, practitioners noted that mental health professionals training should address trauma across a life course, including during pregnancy and the postpartum periods. Practitioners also expressed the importance of services that are informed by cultural humility and holistic care. Holistic care was described to encompass dignity, bodily autonomy, respect, humanity, and empathic care. Creating safe spaces, appropriate client referrals, and dismantling a provider-patient power dynamic were identified as essential solutions and strategies for trust-building and responding to client's needs.

In providing culturally congruent care, practitioners noted that the mental health workforce needs acceptable and accessible decolonized practitioners, who are transparent about historical trauma, relations and their own professional and institutional impact on the population being served (10). Stakeholders indicated that their clients wanted to receive care from providers who looked like them and could relate to their lives. Cultural congruence enables patients to trust their provider's shared lived experience and allows the provider to pinpoint the important elements of the client's experience in life for better diagnosis and treatment. For this, stakeholders emphasized the importance of investing in Black women mental health professionals, including birth workers, social workers, psychologists, and therapists. In addition to investing in culturally congruent practitioners of the next generation, stakeholders also emphasized the importance of investing in Black-women-led organizations that serve Black communities. Practitioners identified that funding should be prioritized for those organizations that commit to advancing Black maternal health for their infrastructure and capacity-building. This shift in funding allocation would allow organizations to meet the needs of their communities.

Several practitioners described the need for both workers to improve Black women's experiences in seeking and receiving maternal and maternal mental health services in disjointed health care settings. Black women and birthing people successfully navigate the mental health care systems by having advocates such as doulas and midwives. Practitioners emphasized the need to strengthen mental health care providers, advocates, and birth worker's partnerships for more accessible health care services and shared decision making. These five pathways emphasize the importance of investing in equitable and antiracist maternal health care to to optimize infant health and wellness.

## Discussion

The results from this research are grounded in the belief that pregnant and parenting people's rights, safety, and dignity are deeply tied to the wellbeing of infants and children. The objective is to continue an interdisciplinary dialogue around the ways in which reproductive injustices harm infant mental health and to encourage further consideration of what this means for infant mental health clinical practices. Investing in maternal mental health could have a transformative impact on infant mental health as it emphasizes the importance of the intersecting oppressions that shape the lives and wellbeing of infants for the rest of their lives. The results further suggest that clinicians should consider collective dialogue, organizing, and action between a multidisciplinary stakeholder group of maternal mental health and infant mental health practitioners, researchers, and individuals with lived experience.

Participants noted the importance of dismantling the false premise that Black women and birthing people are solely responsible and should bear the burden of adverse infant health outcomes and experiences. Social justice frameworks that situate inequities within social and structural systems of care help to reframe the narrative about how we collectively address maternal and infant mental health inequities.

### Implications

Using Reproductive Justice and birth equity as theoretical frameworks and praxis to address perinatal mental health and wellness leads to a broadening of the scope of awareness, practice, and intervention for equitable antiracist mental healthcare for Black mothers, birthing people, and their families. To further unravel the complex challenges of Reproductive Justice and maternal mental health in infant mental health, case studies and cross-cultural comparative studies should be employed. Case studies in infant mental health would serve as a method to increase awareness and identify and address injustices and trauma. While cross-cultural studies would continue to direct the trajectory of developing services (14), including the lived experience of those most affected by trauma, gender oppression, sexism, and racism to be involved in program development and evaluation of the impact of services received, assures understanding and improvement of future practice (15, 16).

Five key pathways generated from this analysis included: (a) Educating and training practitioners, (b) Investing in Black women mental health workforce, (c) Investing in Black women-led community-based organizations, (d) Valuing, honoring, and investing in the community and, (e) Promoting integrated care and shared decision making. The five key pathways identified by Black women mental health stakeholders present key areas of focus to address barriers that impede access to maternal mental health care, but also strengthen community-systems of care and strategies that Black women and birthing people adopt to meet their mental health needs. Educating and training practitioners in how systems of oppression (e.g., racism, gender oppression, and classism) impact pregnant and postpartum people's perinatal experiences position them to be more effective in meeting their client's needs. Participants noted that Black women and birthing people often neglect their own needs to prioritize the needs of their families. These are the same systems that mental health practitioners are indoctrinated into which perpetuates the devaluation of Black women and Black bodies. Black women and birthing people want and deserve to see themselves reflected in the practitioners that care for them. Relatedly, Black women and birthing people are best equipped to fill in gaps that exist within their communities to prioritize healthy and fulfilling perinatal experiences. Sustainable investments in Black women mental health workforce and Black women-led community-based organizations is critical. Traditional healing practices and approaches that support community cohesion are foundational to effective strategies for improved health and wellness for Black people. Postpartum doulas were referenced as components of potential solutions to provide support during a period in which many women and birthing people are vulnerable to developing or exacerbating a mental health condition. Lastly, the fractures in health care systems create barriers for accessing care and lack of shared decision making disempowers birthing people from fully and authentically actualizing their needs and desires.

### Limitations

This study had several limitations. First, the bulk of the interview questions in the facilitators guide focused on maternal mental health, consequently we obtained less insight and resolution specific to infant mental health. Second, the pathways identified centered Black mothers, not capturing the specific needs of non-binary Black birthing populations. Third, the practitioners interviewed identified as cisgender Black women, with a majority of their population served identifying as cisgender Black women, despite recruitment for participants that provided care to Black mothers and birthing people. Lastly, limitations occurred during the recruitment process and strategy. The bulk of our recruitment was based on personal reference, and meeting criteria.

## Conclusion

Instituting education on healthy and culturally appropriate ways to support infant development in parent education programs may support Black parents in establishing healthy attachment and bonds. Prioritizing strategies to improve maternal mental health and centering Black parents in the development of these educational parenting programs may optimize parenting experiences and infant's health, development, and wellness.

## Data Availability Statement

The datasets presented in this article are not readily available because our IRB does not allow for data sharing with external researchers. Requests to access the datasets should be directed to IM, imorgan@birthequity.org.

## Ethics Statement

The studies involving human participants were reviewed and approved by Institutional Review Board of the Institute for Women and Ethnic Studies. The patients/participants provided their written informed consent to participate in this study.

## Author Contributions

IM, TE, and KM conceived the idea for the study and conceptualized the pathways from the data of the key stakeholders. KM facilitated interviews with TE or IM present to take notes for each interview. TE and IM were responsible for conducting a rapid and in-depth analysis of the raw data from the interviews. TE drafted the manuscript. IM, KD, and JC revised the manuscript for important intellectual content. All authors contributed to the article and approved the submitted version.

## Funding

This project was supported by funding from the Perigee Fund.

## Author Disclaimer

The findings and conclusions in this report are those of the authors and do not necessarily represent the official position of Perigee Fund.

## Conflict of Interest

The authors declare that the research was conducted in the absence of any commercial or financial relationships that could be construed as a potential conflict of interest.

## Publisher's Note

All claims expressed in this article are solely those of the authors and do not necessarily represent those of their affiliated organizations, or those of the publisher, the editors and the reviewers. Any product that may be evaluated in this article, or claim that may be made by its manufacturer, is not guaranteed or endorsed by the publisher.
