# Supplementary material for: Black Perinatal Mental Health: Prioritizing Maternal Mental Health to Optimize Infant Health and Wellness
Source: Front Psychiatry. 2022 Apr 29;13:807235. doi: 10.3389/fpsyt.2022.807235 (PMC9098970; doi:10.3389/fpsyt.2022.807235)
Supplement: Supplementary file 1 [file Table_1.pdf]

Table S1. Five Pathways and Strategies Toward Equitable Maternal Mental Health and Infant Health and Wellbeing

	Pathway	Interpretation	Supporting Quote
1.	Investing in Black women led community based organizations	Increase and expand funding for organizational infrastructure and capacity-building (e.g., funding full-time staff, supporting organizational expansion and growth) by investing in organizations to support comprehensive case management that is inclusive of addressing trauma, and social determinants.	"So my thing with the funding is making sure it's going to the right organizations, because just because something is very white-washed and it looks fun and happy and, 'Let's put some Black faces on it' ... I've noticed that during the pandemic. 'Let's just put a Black face and a Black mom and let's make it happy because the funding is there.' But people see that and they notice it, so I just feel like, for those small organizations that are in our communities working so much and every day, it's so many barriers for us to even break down to get the appropriate funding."- Participant #5, Licensed Mental Health Therapist
2.	Educating and training practitioners	Education and training on structural and social determinants of mental health, approaches to addressing trauma, and applying frameworks of cultural humility and respectful care to one's practice.	"I believe the field of psychiatry is predominantly male. And the percentage of black psychologists and psychiatrists is even smaller. How can I go through a system where somebody really doesn't understand me? They don't know what it's like to be a woman. They've never felt what period cramps feels like or having a child. So, not that they can't help form empathy, but you really don't understand." - Participant #1, Perinatal Social Worker<br><br>"I think coming to it with a very deep level of humility and a recognition that it's ... On the universal sense of human rights, it's questionable the way that the systems engage with mothers and babies. A lot of it is around preparing for that process more than it is about helping them prepare to be a mother or helping them. It's just like, how to cope with the system." - Participant #2, Licensed Clinical Psychologist and Birth Doula
3.	Investing in Black women mental health workforce	Develop opportunities (e.g., scholarships and funding mechanisms) for Black women seeking to become mental health practitioners to increase access to culturally congruent and decolonized practitioners	"Diversifying, and let's be real, diversifying don't mean just cause they're Black, or just cause they're brown. Because they went through the same White ass grad program, and they got the same White ass license and work in the same White ass jobs. What I call, these are decolonized practitioners, and that's what my work focuses on…Creating more decolonized practitioners."<br>- Participant #3, Licensed Marriage and Family Therapist<br><br>"I think if birthing people would see more people that looked like them, then they would be more inclined to seek the therapist." - Participant #6, Licensed Mental Health Therapist

4.	Promoting integrated care and shared decision making	Establish a holistic approach to patient care by honoring clients as experts in their body, addressing barriers to postpartum care (e.g., issues of access to mental health care beyond six-weeks postpartum), and developing comprehensive screening tools and referral linkages.	“We were talking to them about the importance of universal screening, not selectively screening. "Oh, you look like you've got issues. You look crazy. Oh, you look together," because that together one's probably the one that's going through it. So what universal depression screening does is it strips that bias. You can't pick and choose who you screen, so everyone must get screened.” Participant #4, Health Disparities Researcher<br><br>“Pregnancy Medicaid ends after a certain amount of time. And so you have all these check-ups when you're pregnant for the baby, but then after you have the baby, it's just like, "Okay, I'll see you at six weeks. And then at six weeks, I'm telling you that you're good enough to continue going on and I'll see you a year later for your pap smear." So that's definitely a barrier of the access that you have when you're pregnant versus the access that you have during the postpartum period.” - Participant #6, Licensed Mental Health Therapist
5.	Valuing, honoring, and investing in community	Decolonize practitioner training and care provision by incorporating traditional healing practices and promoting the use of doulas and midwives in community and health care settings.	“I think, for a lot of us, spirituality is a big thing, and we don't always feel comfortable folding that into the mental health conversation, but it is a part of the conversation. Part of co-creating is ‘What's your spiritual practice? What does it look like? How do you want to incorporate or how do you want to fold it into this process?’ ...But knowing things about that person's practice informs how to help guide them through the most difficult moments, and forms tools that are already available within the practice, that all we have to do is remind them to access. There's a lot of beauty and folding people's spiritual practices into it as well. One of the tougher things can be community, because we shifted from a tribe centered lifestyle to a very individualistic one in this white centered space. I often run across people who will say, ‘I don't have anyone.’ We have to build it.”- Participant #3, Licensed Marriage and Family Therapist
