# Supplementary material for: Black Perinatal Mental Health: Prioritizing Maternal Mental Health to Optimize Infant Health and Wellness
Source: Front Psychiatry. 2022 Apr 29;13:807235. doi: 10.3389/fpsyt.2022.807235 (PMC9098970; doi:10.3389/fpsyt.2022.807235)
Supplement: Supplementary file 2 [file Table_2.pdf]

Table S2. Pathways to Equitable and Anti-Racist Maternal and Infant Mental Health Care

Participant Pseudonym	Training and Expertise
Monica	Perinatal social worker
Felicia	Licensed clinical psychologist; birth doula
Alesia	Health disparities researcher
Yolanda	Licensed clinical psychologist
Michelle	Licensed mental health therapist
Morgan	Licensed mental health therapist
Cassandra	Licensed marriage and family therapist *(specializing in maternal health and maternal-infant bonding)*
Yvette	Birth and postpartum doula
Annette	Licensed clinical professional counselor
Tayler	Certified breastfeeding specialist; parenting support coach
