# Supplementary material for: Black Perinatal Mental Health: Prioritizing Maternal Mental Health to Optimize Infant Health and Wellness
Source: Front Psychiatry. 2022 Apr 29;13:807235. doi: 10.3389/fpsyt.2022.807235 (PMC9098970; doi:10.3389/fpsyt.2022.807235)
Supplement: Supplementary file 3 [file Data_Sheet_1.PDF]

Pathways to Equitable and Anti-Racist Maternal and Infant Mental Health Care

Interview Guide Questions for Stakeholders

SCRIPT

Lead Facilitator: [INTRODUCTIONS - as needed w/ NBEC team]
Observer: [DESCRIBE YOUR ROLE IN NBEC AND ON THE PROJECT AND REASON FOR PARTICIPATING ON THE CALL]

PROJECT OVERVIEW

Lead Facilitator: Thank you for offering your time to participate in this session. The National Birth Equity Collaborative (NBEC) received funding to identify barriers that Black birthing people experience seeking and receiving maternal and infant mental health care services. Additionally, this project seeks to understand how interpersonal and structural racism, sexism, and gender oppression impact access to care, diagnosis, and treatment of Black birthing populations. Ultimately, we are seeking to hold space for conversations that are not being prioritized in maternal and infant mental health. What we learn from this session will be used to inform a roadmap to advance birth equity and reproductive justice in maternal and infant mental health, particularly for Black birthing people.
We anticipate that this session will be approximately 45-55 minutes in duration.

Lead Facilitator: Do you have any questions?

INFORMED CONSENT

Lead Facilitator: We would like to audio record this to ensure that we capture everything that is shared today. We anticipate that a total of five people will have access to audio recordings during the project period. These individuals include myself, two qualitative analysts, and a senior project manager. Lastly, the audio file will be submitted to a transcription service for professional transcription of this session to aid in the analysis of what we learn from these interviews.

The comments that you share today will remain confidential and you will not be identified by any identifying information (such as your name or organization) in any analysis or report. Your participation is completely voluntary, and you are free to end the session at any time. Lastly, you will be compensated with a $200 visa gift card for your participation.

Do we have your consent to record this session?

[IF THE PARTICIPANT GRANTS VERBAL PERMISSION TO RECORD THE SESSION, REQUEST ZOOM TO RECORD THE SESSION TO THE CLOUD.]

!! PRESS RECORD !!

1. Please share with us the **scope of your work** within maternal or infant mental health?

2. Please briefly **describe the population(s) that you serve**.

3. What do you think are the **most important mental health needs** for the Black pregnant and postpartum populations you serve?

4. How does **trauma manifest in the Black birthing populations** that you serve?

Lead Facilitator: The next set of questions asks about barriers. You can think about barriers at the individual, community, or structural level.

5. What are **barriers** that Black women and birthing individuals experience in maternal mental health care? (insurance, providers, access etc)

    a. What are **barriers that Black women and birthing individuals experience in assessing and diagnosing** mental health conditions?

    b. What are **barriers that Black women and birthing individuals experience in accessing** mental health care services?

    c. How do the **barriers** that Black women encounter in accessing maternal health care services **vary during pregnancy vs. during the postpartum period**? *[In this project the postpartum period is defined as the time from the delivery of the placenta to one year post-delivery.]*

Lead Facilitator: The next set of questions asks about racism. You can think about racism at the interpersonal, institutional, or structural levels.

6. How does **racism manifest in Black women and birthing people's experiences** with mental health care diagnosis and treatment? [PROBE: How does **racism impact diagnosis or treatment of mental health conditions?]**

7. How does **sexism and gender oppression manifest in Black women and birthing people's experiences** with mental health care **impact diagnosis or treatment of mental health conditions**? [PROBE: How does **sexism and gender oppression impact diagnosis or treatment of mental health conditions?**]

Lead Facilitator: The next set of questions focus on social determinants that may impact maternal mental health.

8. How does **incarceration, housing, food insecurity, and transportation** affect the populations you serve in seeking care and treatment? How important is case management?
    a. Are there **tools or intake forms that screen for social risk factors** (e.g., food insecurity, housing instability) of patients seeking mental health services?

9. What **Black birthing populations are not visible or uplifted in current maternal mental health discussions**, initiatives and advocacy work?

Lead Facilitator: The next set of questions focuses on solutions and strategies to improve Black women and birthing people's experiences seeking and receiving mental health care.

10. What are ways in which **Black birthing people have successfully navigated mental health care systems**?
    a. What are **examples of strategies that Black birthing people have adopted to improve their mental health** individually or for their communities?

11. How would you describe **anti-racist maternal mental health care**?
    a. What are **strategies or practices that you are aware of that address racism** in maternal mental health?

12. What are **strategies or practices that address sexism and gender oppression** in maternal mental health?

13. What models of care **promote shared decision-making in maternal mental health counseling and treatment**? (formal and/or informal)

14. How would you **describe respectful care in maternal mental health care settings**? [PROBE: What does **respectful care look like?** How does **respectful care make the patient feel?**]

    a. Have you experienced or used **models of care that align with how you described respectful care**?

15. How should **funding be prioritized to improve Black women and birthing people's experiences** seeking and receiving both maternal and mental health care services?

16. How does **culturally appropriate care impact Black women and birthing people's experiences** with mental health care services? (referrals etc..)

17. What would make **mental health care services more accessible and acceptable** to Black birthing populations?

    a. How would **diversifying the mental health workforce** impact Black birthing populations' experiences with mental health care?

    b. How could **healthcare systems better serve communities impacted by food and housing insecurity**?

    c. How could **healthcare systems better serve communities impacted by incarceration and police violence**?

    d. How could **healthcare systems better serve communities impacted by limited transportation**?

Lead Facilitator: The next set of questions focuses on infant mental health.

18. What do you think about when you **hear infant mental health**?
19. How does **maternal mental health impact infant mental health** and the family dynamic?
20. What are **strategies that practitioners can adopt to support parents** in nurturing infant mental health?
21. Is there something that you would like to **share that we have not discussed**?

Last Updated: February 8, 2021
